# Successful treatment of severe myocardial injury complicated with refractory cardiogenic shock caused by AOPP using extracorporeal membrane oxygenation: A case report

**DOI:** 10.1097/MD.0000000000026318

**Published:** 2021-06-11

**Authors:** Yang Li, ChunShui Cao, XiaoLong Luo, Liang Huang

**Affiliations:** Department of Emergency Medicine, The First Affiliated Hospital of Nanchang University, Donghu District, Nanchang, Jiangxi Province, China.

**Keywords:** acute organophosphorus pesticide poisoning, refractory cardiogenic shock, severe myocardial injury, veno-arterial extracorporeal membrane oxygenation

## Abstract

**Rationale:**

Acute organophosphorus pesticide poisoning (AOPP) is a common critical illness observed in clinical practice, and severe AOPP can cause serious cardiac toxicity.

**Patient concerns:**

This patient was a 43-year-old woman who was admitted to the emergency department with acute respiratory failure and hypotension 13 hours after oral consumption of 300 mL of phoxim pesticide.

**Diagnoses:**

Acute organophosphorus pesticide poisoning, cardiogenic shock.

**Interventions:**

We conducted veno-arterial extracorporeal membrane oxygenation (VA-ECMO) therapy as the patient did not respond to conventional measures.

**Outcomes:**

This patient was successfully rescued with VA-ECMO therapy and discharged.

**Lessons:**

We suggest that for patients with severe myocardial injury complicated with cardiogenic shock caused by AOPP, the use of VA-ECMO therapy can improve the prognosis.

## 1 Introduction

Acute organophosphorus pesticide poisoning (AOPP) is one of the most common critical illnesses observed in the emergency department. Annually, approximately 200,000 persons die from AOPP worldwide, and the mortality rate is generally above 15%.^[1]^ AOPP can cause damage to the functions of the heart, lungs, brain, and other systemic organs. Among them, cardiotoxicity mainly manifests as myocardial congestion, interstitial edema, ischemia, and reversible interstitial inflammation.^[2]^ However, when AOPP causes severe myocardial injury with refractory cardiogenic shock, the mortality rate exceeds 60%.^[3]^

The cardiac toxicity caused by AOPP is mostly reversible. Extracorporeal membrane oxygenation (ECMO) can help patients pass through the critical period and lead to a better prognosis. For patients with severe myocardial injury who do not respond to traditional supportive care, the early application of ECMO is indicated.^[4,5]^ We report a case of a middle-aged woman who manifested extensive myocardial injury and a life-threatening hemodynamic disorder after the oral consumption of approximately 300 mL of organophosphorus pesticide. After conventional therapy failed, VA-ECMO was initiated.

## 2 Case report

A 43-year-old woman was admitted to the emergency department with acute respiratory failure and hypotension 13 hours after oral consumption of 300 mL of phoxim pesticide. She had no history of heart disease. The physical examination on admission showed the following: temperature, 36.8°C; heart rate, 84 beats/min; respiratory rate, 17 beats/min; and blood pressure, 81/42 mm Hg (norepinephrine, 0.2 μg/kg/min). She had clear consciousness, the pupils on both sides were equal and 3.5 mm in diameter, the skin was dry all over the body, there were no wet rales over both lungs on pulmonary auscultation, and there was no murmur on heart auscultation. The arterial blood gas analysis and myocardial enzyme spectrum were normal, while electrocardiography (ECG) showed sinus tachycardia on admission, and admission echocardiography showed that the motion of each segment of the left ventricular wall declined diffusely, and the left ventricular ejection fraction was 35%. Bedside chest X-ray (CXR) revealed abnormal density of both lungs, considering pulmonary edema or changes after poisoning. After admission, the patient was intubated and mechanically ventilated, analgesia and sedation were administered, rapid fluid expansion combined with norepinephrine was administered as an anti-shock treatment, hemoperfusion was performed, and atropine was administered to reverse the inhibitory effects of AOPP on cholinesterase.

**Figure 1:**
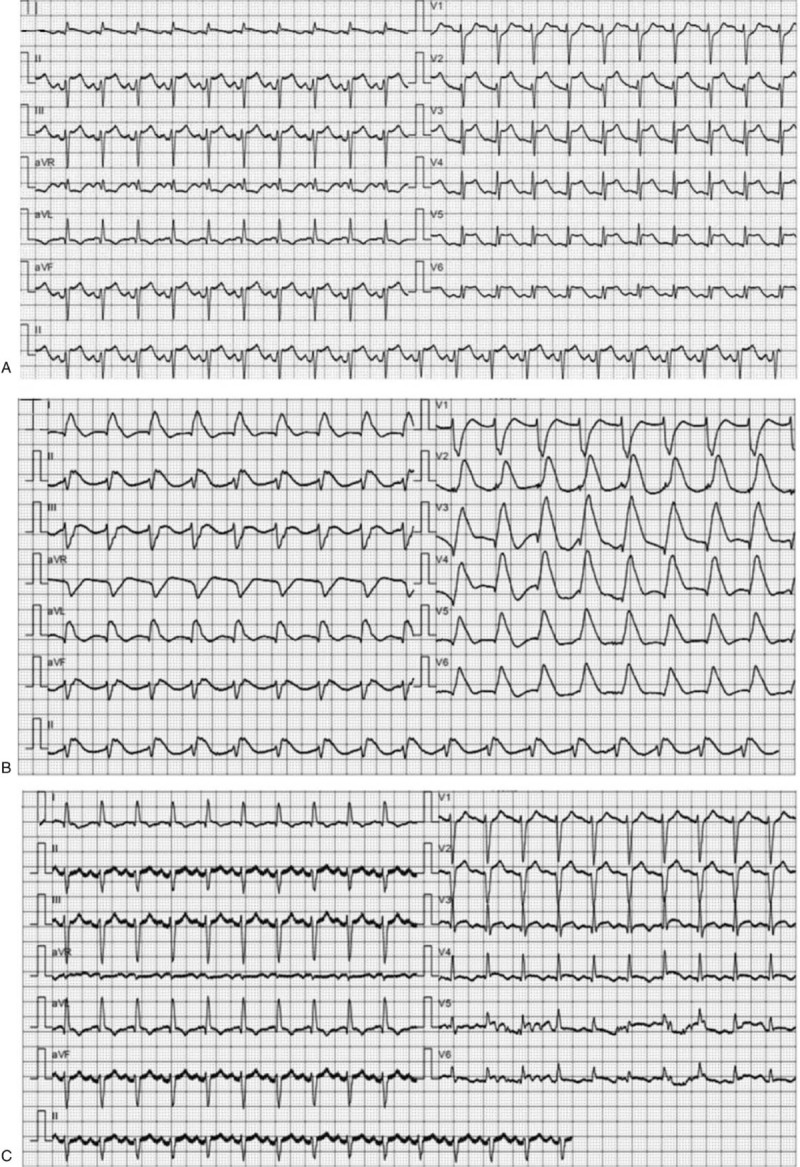
Panel A: Twenty three hours after admission, the ECG showed extensive lead ST segment elevation. Panel B: The emergency ECG revealed ventricular arrhythmia 34 hours after admission. Panel C: The ECG showed a decrease in the ST segment 3 hours after VA-ECMO was performed.

Twenty three hours after admission, the ECG (Fig. 1 A) showed extensive lead ST segment elevation. The patient progressively deteriorated over the 36 hours after admission, and the ECG showed ventricular arrhythmia with abnormal Q wave and ST-T segment changes in some leads (Fig. 1 B), which indicated severe myocardial injury. Echocardiography revealed an ejection fraction of 30%, and arterial blood gas analysis showed that the lactate level was 12.5 mmol/L. The laboratory results showed a notable cTnI level of 11.0 μg/L (reference range, 0.01–0.023); the NT-ProBNP level was 26700 ng/L (reference range, 300–450), the creatine kinase level was 2454 U/L (reference range, 40–200), and the creatine kinase-MB isoenzyme level was 166 U/L (reference range, 0–24). She subsequently developed cardiogenic shock due to severe myocardial injury with complications of ventricular arrhythmia; thus, VA-ECMO was immediately initiated.

We used the Seldinger technique, and percutaneous ultrasound-guided femoro-femoral access was chosen for peripheral VA-ECMO configurations, which involve blood drainage from femoral venous access and reinfusion through a femoral arterial cannula. A distal perfusion cannula in the ipsilateral superficial femoral artery was also placed. The initial blood flow rate was 4.5 L/min while an oxygen flow rate of 4 L/min, and 5000 units of heparin were intravenously administered at the time of ECMO initiation. The heparin dosage was adjusted to maintain an activated clotting time (ACT) of 180 to 220 seconds. Monitoring of anticoagulation was performed according to the protocol and maintained by recording platelet counts, INR, and APTT daily.

Three hours after the initiation of ECMO, the ECG showed a decrease in the ST segment (Fig. 1 C). Her hemodynamics improved 6 hours after the initiation of ECMO. On the 3rd day after the initiation of ECMO, the myocardial enzyme indicators decreased significantly, and the left ventricular ejection fraction was 46%. VA-ECMO was withdrawn. On the 11th day after admission, she was transferred out of the intensive care unit, and at the 25-day follow-up, the patient had recovered and was discharged. The only complication during hospitalization was local bleeding at the puncture site.

## 3 Discussion

Phoxim is an organophosphorus pesticide that can inhibit cholinesterase activity. Oral administration of Phoxim in large doses often causes severe cardiac toxicity. Chen^[1]^ et al found that 52.0% of AOPP patients developed acute myocardial injury. The main mechanisms underlying myocardial injury may be as follows:

1. direct toxic effects on the myocardium by pesticides, solvents, impurities, etc.;
2. sympathetic and parasympathetic dysfunction, the release of a large quantity of catecholamines and increased sensitivity of the heart to catecholamines, causing coronary artery spasm, myocardial ischemia, and myocardial injury;
3. the accumulation of a large amount of acetylcholine and the release of a large quantity of cytokines and inflammatory mediators affecting myocardial cells and resulting in toxic myocarditis; and
4. complications of respiratory failure, severe electrolyte disturbances, and acidosis causing internal environmental disturbances and aggravating myocardial damage.^[2,6,7]^

In addition, Anand^[8]^ et al found that the pathology of myocardial injury in patients with AOPP was characterized by myocardial interstitial edema, vascular congestion and inflammatory changes in the myocardium. Severe myocardial injury due to AOPP often leads to various life-threatening cardiac arrhythmias, which are the main cause of sudden death.

As a bridge to recovery from cardiopulmonary failure, ECMO can improve the survival rate of patients with refractory cardiogenic shock following poisoning.^[9,10]^ Vardon^[11]^ et al reported the successful rescue of patients with intractable ventricular arrhythmia and cardiogenic shock caused by severe yew poisoning using VA-ECMO. In addition, ECMO was used in patients with severe aluminum phosphide poisoning, reducing the mortality rate from 84.4% to 40%.^[3]^ In addition, in a retrospective cohort analysis of persistent cardiac arrest or severe shock caused by drug poisoning, Masson^[12]^ et al found that the survival rate of patients who received ECMO was as high as 86%, while that of patients who did not receive ECMO was only 48%. Furthermore, in a meta-analysis of the application of ECMO in patients with severe cardiac damage caused by drug poisoning, the survival rate was as high as 66%.^[13]^ Thus, ECMO can be used as an effective therapeutic option for patients with severe poisoning.

The successful use of ECMO in patients with severe cardiotoxicity due to poisoning with various drugs has been reported. However, reports of the utilization of ECMO in patients with cardiogenic shock caused by AOPP are rare. We used ECMO to successfully rescue a patient with severe myocardial injury caused by AOPP associated with refractory cardiogenic shock and fatal arrhythmia. ECMO serves as a bridge to recovery in patients with severe poisoning, allowing the injured myocardium to rest. With the removal of organophosphorus pesticides, cardiac function can be restored by the application of ECMO.

AOPP can cause fatal cardiac injury. This case suggests that when patients do not respond to conventional therapy, the early use of ECMO is an effective therapeutic option for refractory cardiogenic shock caused by AOPP, and more data are needed to verify its effectiveness.

## Author contributions

**Writing – original draft:** Yang Li, Chunshui Cao, Xiaolong Luo.

**Writing – review & editing:** Liang Huang.
